# Airway Epithelial Innate Immunity

**DOI:** 10.3389/fphys.2021.749077

**Published:** 2021-11-26

**Authors:** Sebastian L. Johnston, David L. Goldblatt, Scott E. Evans, Michael J. Tuvim, Burton F. Dickey

**Affiliations:** ^1^National Heart and Lung Institute, Imperial College London, London, United Kingdom; ^2^Department of Pulmonary Medicine, University of Texas MD Anderson Cancer Center, Houston, TX, United States; ^3^University of Texas Rio Grande School of Medicine, Edinburg, TX, United States; ^4^Howard Hughes Medical Institute, Chevy Chase, MD, United States

**Keywords:** airway, epithelium, innate immunity, immunity, mucus

## Abstract

Besides providing an essential protective barrier, airway epithelial cells directly sense pathogens and respond defensively. This is a frontline component of the innate immune system with specificity for different pathogen classes. It occurs in the context of numerous interactions with leukocytes, but here we focus on intrinsic epithelial mechanisms. Type 1 immune responses are directed primarily at intracellular pathogens, particularly viruses. Prominent stimuli include microbial nucleic acids and interferons released from neighboring epithelial cells. Epithelial responses revolve around changes in the expression of interferon-sensitive genes (ISGs) that interfere with viral replication, as well as the further induction of interferons that signal in autocrine and paracrine manners. Type 2 immune responses are directed primarily at helminths and fungi. Prominent pathogen stimuli include proteases and chitin, and important responses include mucin hypersecretion and chitinase release. Type 3 immune responses are directed primarily at extracellular microbial pathogens, including bacteria and fungi, as well as viruses during their extracellular phase of infection. Prominent microbial stimuli include bacterial wall components, such as lipopeptides and endotoxin, as well as microbial nucleic acids. Key responses are the release of reactive oxygen species (ROS) and antimicrobial peptides (AMPs). For all three types of response, paracrine signaling to neighboring epithelial cells induces resistance to infection over a wide field. Often, the epithelial effector molecules themselves also have signaling properties, in addition to the release of inflammatory cytokines that boost local innate immunity. Together, these epithelial mechanisms provide a powerful first line of pathogen defense, recruit leukocytes, and instruct adaptive immune responses.

## Introduction

### Barrier Properties of the Airway

The most basic protective function of surface epithelial cells is to provide a structural barrier. This both hinders the entry of pathogens, noxious chemicals, and physical objects into interior tissues, and prevents the egress of interstitial fluids. The epithelial barrier of the conducting airways of the lungs is comprised mostly of a surface mosaic of ciliated and secretory cells, with small numbers of scattered neuroendocrine cells, tuft cells, and ionocytes (Zaragosi et al., 2020). These surface epithelial cells are physically tied together by multiple layers of intercellular junctions that connect to intracellular cytoskeletal proteins (Frey et al., 2020). In proximal intrapulmonary airways, the epithelium is pseudostratified, with basal cells serving as progenitors. In distal airways, the epithelium has a simple single-layer configuration without basal cells. Normally, the airway epithelium is highly stable, with surface cells having a life span of ∼1 year. However, following injury, airway epithelial cells show great plasticity in maintaining a continuous structural barrier, flattening and losing their normal differentiated phenotypes to quickly cover the surface (Park et al., 2006). Basal cells in proximal airways proliferate vigorously to repopulate a normal surface epithelium, and differentiated cells in distal airways can reenter the cell cycle to proliferate, particularly the secretory cells (Chen, 2017). While maintenance of a structural barrier is a core epithelial function, increased epithelial permeability can also be an important defense against infection by allowing the passage of antimicrobial plasma proteins into the airway lumen, and can contribute pathologically to allergic airway disease by both allowing entry of allergens into tissue and pro-inflammatory plasma proteins into the airway lumen (Frey et al., 2020).

**FIGURE 1:**
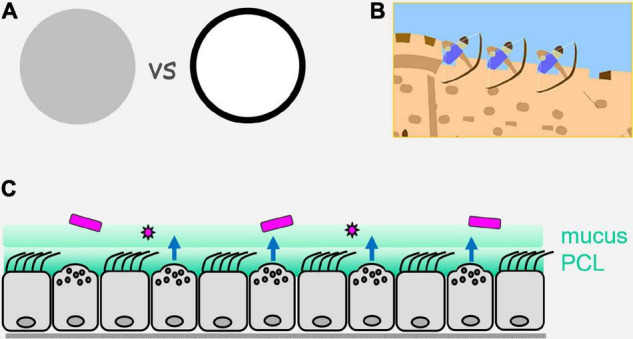
Perimeter defense. **(A)** On the left is illustrated an army or a multicellular organism with sentinels and defenders distributed evenly (uniform gray). On the right is illustrated the concentration of sentinels and defenders at the perimeter (black), with a paucity of sentinels and defenders in the center (white). Advantages of the perimeter defense are that by placing sentinels that have limited range (e.g., direct vision for military sentinels, and local diffusion of pathogen molecules for epithelial sentinels) at the border, they are best positioned for the earliest detection of invaders; by placing defenders that have limited range (e.g., archers for military defense, and reactive oxygen species for epithelial defense) at the perimeter, they are best positioned to engage invaders. **(B)** Illustration of a castle wall as a physical barrier that can itself impede invasion, and can also allow the placement on its structure of sentinels and defenders that are ideally positioned to detect and actively repulse invaders. **(C)** Illustration of the airway epithelium as a mosaic of ciliated and secretory cells that form a physical barrier containing innate pathogen sensing mechanisms and active defenses operating over short distances. Pathogens are shown in purple, an overlying mobile mucus layer and a dense and immobile periciliary layer (PCL) are shown in green, and active defenses are shown as blue arrows that are elaborated in Figure 2.

Surface epithelial cells throughout the body display a variety of specialized mechanisms to provide an effective barrier, including cornification of the skin, and production of a thick layer of adherent mucus in the gastrointestinal tract. In the intrapulmonary airways, a non-adherent layer of mucus is secreted from surface epithelial cells and glands, then propelled from distal to proximal airways and out of the lungs by ciliary beating to clear inhaled particles, pathogens and toxicants (Knowles and Boucher, 2002; Fahy and Dickey, 2010). By this highly effective steady-state mechanism of mucociliary clearance, many external threats never even contact the underlying epithelium (Figure 1). The importance of this mobile barrier is emphasized by the lung inflammation and infection that develop in mice and humans with deficiency of mucus production or ciliary beating (Roy et al., 2014; Horani and Ferkol, 2021); the positive selection in humans of an overexpressing allele of the constitutively expressed mucin MUC5B, which presumably helps protect against infection even though it results in pulmonary fibrosis late in life (Evans et al., 2016); and constraint against monoallelic loss-of-function of MUC5B in humans (Lek et al., 2016). This mobile mucus layer is transported over a dense periciliary layer comprised of membrane-bound mucins and other glycoconjugates (the “glycocalyx”) that provides another important level of defense (Fahy and Dickey, 2010; Dickey, 2012; Frey et al., 2020).

### Inducible Resistance to Infection of Airway Epithelium

**FIGURE 2:**
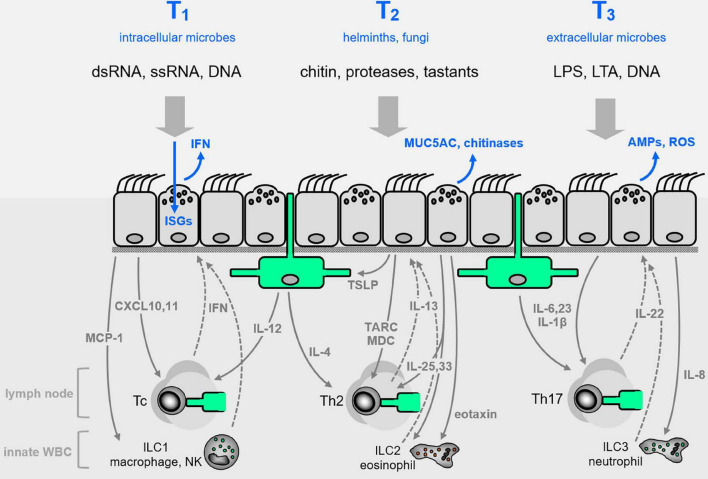
Airway epithelial innate defenses. The airway epithelium illustrated in Figure 1C is elaborated here, with representative pathogen-associated molecular patterns (PAMPs, black lettering near top) that signal the presence of pathogen classes evoking characteristic Type 1, 2, or 3 (T1, T2, T3) innate immune responses (blue lettering at top). Direct epithelial anti-infective responses are shown in blue within or directly above the epithelium. Intercalated among the epithelial cells are dendritic cells shown in green, and below the epithelium are shown other leukocytes, as well as cytokines that signal between epithelium and leukocytes (with signals from epithelium to leukocytes as solid gray arrows, and signals from leukocytes to epithelium as dashed arrows). See text for abbreviations.

For many years, the barrier properties of surface epithelial cells were thought to be their only contribution to organismal defense. However, with a focus for the past 30 years on the innate immune system came recognition of the capability of epithelial cells to sense pathogens and mount direct antimicrobial effector responses, as well as to recruit leukocytes (Evans et al., 2010; Whitsett and Alenghat, 2015; Frey et al., 2020). In lower metazoans that have few or no leukocytes, the expression of innate receptors is concentrated in surface epithelial cells (Tzou et al., 2000; Evans et al., 2010). This strategy makes sense because initial contact with pathogens takes place at tissue surfaces, so concentrating sensing and effector responses at surfaces maximizes efficiency. Military forces have recognized the value of such a “perimeter defense” for millenia, and that the combination of a physical barrier with active defenses is particularly potent (Figure 1). In higher metazoans, the question arises to what degree the strategy of a strong perimeter defense remained in place during evolution vs. reliance on mobile leukocytes. In the case of mammalian epithelia, the simple answer is that they have retained a strong intrinsic perimeter defense while evolving a complex system of communication with leukocytes (Figure 2). The focus of the remainder of this review is the airway epithelium’s sophisticated repertoire of pathogen detection mechanisms and active defenses. Furthermore, we review how activated epithelial cells signal to neighboring cells to generate an epithelial field with heightened immunity, and how they polarize both their own responses and those of downstream leukocytes to best defend against specific pathogen classes.

## Innate Epithelial Pathogen Sensing and Responding

Immune responses have frequently been divided into three classes, as with innate lymphoid cells (ILCs) that are classified as ILC1, ILC2, and ILC3. Such a tripartite classification is not universally accepted with other leukocyte classes, such as T helper cells, but this scheme seems to work well for understanding epithelial defenses and therefore informs the structure of this review. While threats other than pathogens were mentioned in the previous section, and the clearance of inert particles is an important function of the mobile mucus layer, pathogens powerfully shaped inducible epithelial responses during evolution so are the principle focus of this section. Pathogen-selective epithelial responses were first identified in *Drosophila* (Lemaitre et al., 1997; Tzou et al., 2000). To classify innate epithelial responses in alignment with classification schemes commonly used for leukocytes, we use three criteria: (1) the nature of the stimuli, with pathogen-associated molecular patterns (PAMPs) that evoke polarized leukocyte responses (e.g., ssRNA) expected to similarly evoke polarized epithelial responses, and we cite evidence when available; (2) the effector responses, with molecules associated with polarized responses in leukocytes (e.g., IFN or chitinase expression) similarly associated with polarized epithelial responses; (3) cytokine signaling, with cytokines associated with polarized leukocyte responses (e.g., IFN or IL-4) similarly associated with incoming or outgoing polarized epithelial responses.

### Type 1 Epithelial Immunity

The central feature of Type 1 airway epithelial cell responses is the induction of type I and type III interferons (IFNs) and interferon-stimulated genes (ISGs) (Schoggins, 2019). Type 1 responses are critical to controlling respiratory virus infections, and are also important in controlling intracellular bacteria such as *Mycobacterium tuberculosis* and *Chlamydia* species. However, *Mycobacterium tuberculosis* mostly grows extracellularly during active disease, and resides predominantly in macrophages during dormant infection, so will not be further considered here even though interferons figure prominently in the clearance of macrophage infection. *Chlamydia* are obligate intracellular bacteria, with multiple species capable of infecting airway epithelial cells, most commonly *Chlamydia pneumoniae* (Di Pietro et al., 2019; Gautam and Krawiec, 2021). Manifestations of infection range from asymptomatic to serious, but in aggregate they account for only a small fraction of respiratory infections so will not be further considered here. Instead, viral pathogens are by far the most common cause of pneumonia, bronchitis and bronchiolitis in adults and children (Jain et al., 2015a,b), and are the focus of this section.

#### Sensing Type 1 Pathogen-Associated Molecular Patterns, Damage-Associated Molecular Patterns, and Cytokines

Type I immunity is induced in response to PAMPs and damage-associated molecular patterns (DAMPs) through pattern-recognition receptors (PRRs), which activate antimicrobial and pro-inflammatory responses to effect innate and acquired immunity. The major families of PRRs involved in viral sensing are the Toll-like receptors (TLRs), which are membrane proteins expressed on cell surfaces and in endosomes, and cytoplasmic PRRs. The latter include, most prominently, the RIG-like helicases (RLHs), the cyclic GMP-AMP synthase—Stimulator of Interferon Genes (cGAS-STING) pathway, and among the large family of NOD-like receptors (NLRs), NLRC2 (Evans et al., 2010; Motta et al., 2015; Ablasser and Chen, 2019). PAMPs that signal virus infections include double-stranded RNA (dsRNA), recognized by TLR3; single-stranded RNA (ssRNA), recognized by TLR7, TLR8, and NLRC2; and DNA, particularly unmethylated CpG motifs, recognized by TLR9. These signatures are also recognized by retinoic-acid inducible gene-I (RIG-I), coded by *DDX58*, and melanoma differentiation-associated 5 (MDA5), coded by *IFIH1*. RIG-I and MDA5 interact with mitochondrial antiviral signaling protein (MAVS) on the outer surface of mitochondria, causing MAVS to form aggregates with TNF receptor-associated factor 3 (TRAF3) and I-kappa-B kinase-epsilon/TANK-binding kinase 1 (IKKε/TBK1), leading to the activation of the transcription factors interferon regulatory factor (IRF) 3 and IRF7. These induce the innate anti-viral type I and type III IFNs, IFN-α and IFN-β, and IFN-λ1-4, respectively. STING is an ER-localized protein that acts as a powerful cytoplasmic DNA sensor by binding cyclic GMP-AMP generated by cGAS that directly binds DNA, and as a signaling adaptor for DEAD-Box Helicase 41 (DDX41) and other DNA sensors (Ablasser and Chen, 2019). It activates signal transducer and activator (STAT) 1/2 and IRF3 through TBK1 to strongly induce type I IFN production.

The principle cytokines capable of inducing Type 1 epithelial responses are IFNs released from epithelial cells in an autocrine and paracrine manner, or from leukocytes such as ILC1s, plasmacytoid dendritic cells, and lymphocytes (Schoggins, 2019). DAMPS that promote Type 1 epithelial responses are addressed in the next section.

#### Anti-infective and Maladaptive Type 1 Responses

Virus infection of airway epithelial cells results principally in the induction of the type III IFN-λs and IFN-β, though some, but not all of the 13 subtypes of IFN-α are also induced (Khaitov et al., 2009). The type I IFNs all bind the same surface receptor, composed of two proteins, IFNAR1 and IFNAR2, found on the surface of all nucleated cells (Schreiber, 2017). The type III IFNs signal via their common receptor IFNλR1 (IL28Rα) and IL-10Rβ, which is expressed mostly on epithelial surfaces of the respiratory and gastrointestinal tracts (Galani et al., 2017). Both receptors signal via the Janus family kinases (JAKs) to activate STAT proteins to induce hundreds of ISGs with wide-ranging direct anti-viral activities as well as immune stimulatory activities, including stimulation of adaptive immunity (Schoggins, 2019).

Studies of influenza virus infection in mice show that the IFN-λs are the first IFNs produced, and that they act at the epithelial barrier to suppress initial viral spread without activating inflammation. However, if those initial responses are insufficient and infection progresses, type I IFNs come into play to enhance anti-viral resistance. However, they also induce pro-inflammatory responses, thereby causing immunopathology (Galani et al., 2017). Recent studies in COVID-19 have confirmed that maladaptive IFN responses are associated with severe outcomes. Temporal studies of IFN induction in patients hospitalized with COVID-19 pneumonia showed that IFN-λ and type I IFN production were both diminished and delayed, and were induced in only a small fraction of patients as they became critically ill. Higher IFN-λ concentrations in these patients with COVID-19 correlated with lower viral loads in bronchial aspirates and faster viral clearance, a higher IFN-λ to type I IFN ratio correlated with improved outcomes (Galani et al., 2021), and recovery was paralleled by increased type I IFNs (Contoli et al., 2021).

There are a wide range of intracellular and extracellular DAMPs that may be released during respiratory virus infections, including heat-shock proteins, high-mobility group box 1 (HMGB1), hyaluronan fragments, ATP, uric acid, heparin sulfate, antimicrobial peptides (AMPs), dsRNA, ssRNA and DNA (Roh and Sohn, 2018; Zindel and Kubes, 2020). Mostly they induce the pro-inflammatory cytokines including IL-1β, IL-6, TNF, and CXCL8, which amplify innate and adaptive immune responses. However, one alarmin, IL-33, is notable because it is frequently induced by virus infection of airway epithelial cells and it potently induces Type 2 cytokine production from Th2 cells and ILC2s, resulting in the induction of an asthma phenotype (Jackson et al., 2014; Goldblatt et al., 2020). Type 2 epithelial responses do not seem to induce resistance to respiratory virus infection, rather to the contrary, the Type 2 cytokines IL-4, IL-5, and IL-13 suppress IFN induction (Chen et al., 2014; Contoli et al., 2015; Gielen et al., 2015; Hatchwell et al., 2015), so it is unclear whether the induction of IL-33 by respiratory viruses is a viral immune evasion mechanism or there is some adaptive value to the host that is not currently recognized.

#### Therapeutic Potential of Modulating Type 1 Responses

Many studies have reported delay and deficiency in induction of type I and type III IFNs in response to virus infection of airway epithelial cells in asthma (Wark et al., 2005; Contoli et al., 2006), and these delays and deficiencies are implicated in increased severity of virus-induced exacerbations (Contoli et al., 2006). Similar studies report that IFN deficiency is implicated in increased chronic obstructive pulmonary disease (COPD) exacerbation frequency (Singanayagam et al., 2019). These studies led to a clinical development program to study the effect of inhaled IFN-β on worsening of asthma symptoms caused by viral infections (Djukanovic et al., 2014; McCrae et al., 2021). These studies reported encouraging improvements in symptom severity and lung function in people with moderate/severe asthma, but neither study met its primary endpoint as insufficient numbers of volunteers suffered virus-induced worsening of their asthma of sufficient severity for the treatment to have an impact.

IFN therapy has also been studied recently in COVID-19, with early subcutaneous IFN-β proving effective at reducing severe outcomes, and early subcutaneous IFN-β and IFN-λ both proving effective at speeding up virus clearance when administered in the first 7 days from symptom onset (Hung et al., 2020; Feld et al., 2021). Inhaled IFN-β was also reported effective at speeding up recovery of patients admitted to hospital with COVID-19 symptoms (Monk et al., 2021).

### Type 2 Epithelial Immunity

Airway epithelial cell Type 2 responses are central in controlling multicellular parasite (helminth) migration through the lungs, in the response to fungal infection of the airways, and in the pathogenesis of allergic asthma. Similarities in clinical presentation between parasite infections of the lungs and allergic asthma have long been noted, but a molecular relationship between the two was only established in recent decades. In particular, IL-13 and its upstream and downstream signaling pathways have been demonstrated to be essential both for resistance to parasites and for full expression of the allergic asthma phenotype (Frey et al., 2020; Weatherhead et al., 2020; Hammad and Lambrecht, 2021). Upregulated expression of the secreted mucin MUC5AC is a prominent effector response in both clinical settings (Fahy and Dickey, 2010). Like helminth infestations, fungal infections of the airways also prominently evoke Type 2 epithelial responses. Historically, this was first recognized in allergic bronchopulmonary aspergillosis (ABPA), but recently, infection by various other fungi has been found to be a frequent driver of allergic asthma, and Type 2 epithelial responses have been demonstrated to be fungistatic, cementing the relationship between fungal infection and Type 2 responses (Millien et al., 2013; Bigot et al., 2020). Mechanisms that underlie Type 2 epithelial immunity and their therapeutic manipulation are the focus of this section.

#### Sensing Type 2 Pathogen-Associated Molecular Patterns, Damage-Associated Molecular Patterns, and Cytokines

Many of the PAMPs that signal microbial infection by viruses and bacteria do not figure prominently in the recognition of infection by parasites or fungi. Thus, PRRs such as the TLRs and RLRs that are important in Type 1 and Type 3 responses are not usually involved in the initiation of Type 2 responses. An important exception is the recognition of allergic stimuli by TLR4 through the binding of fibrinogen cleavage products or by functional mimicry of the TLR4 binding protein MD-2 by allergens (Hammad et al., 2009; Trompette et al., 2009; Millien et al., 2013). Proteolytic activity is a common feature of allergens, and the release of extracellular proteases is also a prominent feature of fungal growth and parasite invasion (Li et al., 2021). Fibrinogen cleavage products directly upregulate MUC5AC production in isolated airway epithelial cells (Millien et al., 2013), and protease-activated receptors (PARs) also sense the presence of pathogens and allergens (Li et al., 2021).

An important molecular component of both helminths and fungi that signals infection is chitin, which is found in the cuticle and grinder of multiple helminths, and in the cell wall of multiple fungal species (Dickey, 2007; Przysucha et al., 2020). Introduction of chitin into the lungs of mice strongly induces airway epithelial mucous metaplasia and the accumulation of ILC2s and eosinophils (Reese et al., 2007). Mechanisms of the sensing of chitin in the airway have not been fully determined, but likely involve recognition by chitinases and chitinase-like proteins. In turn, upregulated expression of chitinases and chitinase-like proteins is a prominent feature of Type 2 responses. Other fungal cell wall components that activate Type 2 responses are β-glucans, galactomannans, and mannoproteins, which signal via C-type lectin-type receptors (CLR) on epithelial cells directly or indirectly through leukocytes (Li et al., 2021). Tuft cells are a rare cell type in the airway that express receptors for succinate and tastants, leading to the release of eicosanoids and IL-25 that strongly induce Type 2 responses (Schneider et al., 2019).

Multiple cytokines communicate between leukocytes and lung parenchymal cells in the development of Type 2 immune responses, but among these, IL-13 plays a central role in epithelial responses (Frey et al., 2020; Hammad and Lambrecht, 2021). It is capable of inducing many or all of the epithelial effector responses described in this section, and conversely, inhibition of IL-13 results in incomplete responses to most other stimuli.

#### Anti-infective and Maladaptive Type 2 Responses

While the pathologic role of mucus hyperproduction and rapid secretion (together termed “mucus hypersecretion”) in fatal asthma has been recognized for more than a century (Fahy and Dickey, 2010), the protective role of mucus hypersecretion in resistance to parasite migration through the lungs has only been demonstrated recently (Campbell et al., 2019). Specifically, lumenal mucus traps larvae in the airway lumen, preventing the completion of the parasite life cycle of migration to the gut. Similarly, chitinases and chitinase-like proteins promote resistance to both parasites and fungi, but can also contribute to the pathophysiology of asthma and other airway diseases (Bigot et al., 2020; Przysucha et al., 2020). The roles of multiple other molecules involved in Type 2 epithelial responses have a similar two-sided character, participating both in resistance to helminths and fungi and in the pathogenesis of non-infectious airway diseases (Hammad and Lambrecht, 2021).

#### Therapeutic Potential of Modulating Type 2 Responses

In much of the world, parasite infestation remains a major problem. However, in industrialized countries, parasite infection is rare whereas allergic diseases are a greater problem. Thus, therapies targeting Type 2 signaling, such as monoclonal antibodies directed against IL-13, IL-5, IL-33, TSLP (thymic stromal lymphopoietin) or their receptors, or against IgE, have been effective in treating allergic asthma without inducing a substantial cost in parasite clearance (Hammad and Lambrecht, 2021). Neither have these therapies been associated with an increase in airway fungal infection, though whether this is due to the effectiveness of baseline mucociliary clearance, the effectiveness of Type 3 antifungal immune mechanisms that act in parallel to Type 2 mechanisms, or some other reason, is not clear. Targeting Type 2 effector mechanisms, such as chitinases, MUC5AC, and ClCa (chloride channel accessory) signaling in allergic diseases is also being explored (Keeler et al., 2018; Przysucha et al., 2020), and might be expected to have similar benefit without incurring substantial cost in parasite or fungal defense. By the same line of reasoning but in the opposite direction, augmentation of Type 2 epithelial immune responses would generally not be expected to have therapeutic value in industrialized countries.

### Type 3 Epithelial Immunity

Airway epithelial cell Type 3 responses are central to killing extracellular bacteria, and are also capable of killing fungi and inactivating viruses during the extracellular phase of their infectious cycle. In the elucidation of innate immune mechanisms during the last century, Type 3 responses were the first studied, reflecting the initial experimental challenges of insects with bacteria and fungi (Lemaitre et al., 1997; Tzou et al., 2000). That work led to the recognition of the role of Toll in the sensing of PAMPs, and the subsequent identification of TLRs in mammals. AMPs were the first effectors studied in insects (Lemaitre et al., 1997; Tzou et al., 2000), but further work revealed a critical role for the generation of reactive oxygen species (ROS) by gut epithelium in defense against pathogenic bacteria (Ha et al., 2005). In mammals, activation of Type 3 epithelial immunity evokes vigorous neutrophil recruitment, which also plays an important role in control of infection by extracellular microbes as indicated by the susceptibility of neutropenic human subjects to bacterial and fungal infections.

#### Sensing Type 3 Pathogen-Associated Molecular Patterns, Damage-Associated Molecular Patterns, and Cytokines

PAMPs that signal bacterial infections include bacterial lipopolysaccharide (LPS), endotoxins found on the cell membranes of Gram-negative bacteria (recognized by TLR4), bacterial flagellin (recognized by TLR5), and lipoteichoic acid and peptidoglycans from Gram-positive bacteria (recognized by TLR1/2/6). NLRs similarly recognize many of the same PAMPs, and activation of both TLRs and NLRs by the same stimulus can promote particularly vigorous responses. Besides these well-recognized PRRs, numerous other epithelial receptors sense the presence of microbes directly or indirectly, such as CLRs and complement receptors (Evans et al., 2010; Motta et al., 2015; Ablasser and Chen, 2019).

Many DAMPs also activate Type 3 responses in the recognition of tissue injury and cell death (Roh and Sohn, 2018; Zindel and Kubes, 2020). These include macromolecules released from extracellular matrix such as hyaluronan and decorin, proteins released from the cytoplasm such as heat shock and S100 proteins, proteins released from the ER such as calreticulin, proteins released from mitochondria such as formyl peptides and mitochondrial transcription factor A (TFAM), and nucleic acids and proteins released from the nucleus such as HMGB1 and IL-1α. Many of these activate TLRs and NLRs, and they may also activate distinct receptors such as the receptor for advanced glycation endproducts (RAGE) and formyl peptide receptor 1 (FPR1). Besides these macromolecules, small molecules such as ATP and uric acid can signal tissue damage via purinergic receptors and NLRs.

Cytokines that mimic PAMPs and DAMPs in activating Type 3 epithelial immune responses are IL-22 and multiple IL-17 family members (Dudakov et al., 2015; Swedik et al., 2021). IL-22 and some IL-17 family members are secreted by leukocytes to activate epithelial cells, while other IL-17 family members are secreted by epithelial cells in response to PAMPs and DAMPs to signal to neighboring epithelial cells and to leukocytes.

#### Anti-infective and Maladaptive Type 3 Responses

Historically, most attention on Type 3 responses was focused on neutrophil recruitment, induction of inflammatory cytokines such as IL-1β, IL-6, and TNF, and the induction of AMPs. However, as noted above, ROS generation by epithelial cells was identified early as a critical antimicrobial defense in *Drosophila* (Ha et al., 2005). In the mammalian airway, antimicrobial resistance can be powerfully induced by exposure to an aerosolized bacterial lysate or to the combination of TLR agonists ODN M362 (agonist of TLR9 and multiple cytoplasmic nucleic acid sensors) and Pam2CSK4 (agonist of TLR2/6) (Evans et al., 2011), as well as other PRR ligands (Evans et al., 2010), or IL-22 or IL-17 cytokines (Dudakov et al., 2015; Swedik et al., 2021). Recently, the principal effector of the resistance response to ODN-Pam2CSK4 was found to be ROS generated both by DUOXs (dual oxidases) and mitochondria (Kirkpatrick et al., 2018; Ware et al., 2019). Thus, ROS generation appears to be a central defense mechanism from flies to mammals. A multipronged airway epithelial response is characteristic of Type 3 immunity, with cooperation among the limbs of the response as exemplified by secretion of both ROS and the antimicrobial protein lactoperoxidase, resulting in the generation of highly potent antimicrobial oxidized halides (Kirkpatrick et al., 2018; Ware et al., 2019; Lazzaro et al., 2020; Yamakaze and Lu, 2021). In a maladaptive context, all limbs of the response—neutrophils, inflammatory cytokines, AMPs, and ROS—are thought to contribute to the pathogenesis of airway diseases including asthma, COPD, and bronchiectasis.

#### Therapeutic Potential of Modulating Type 3 Responses

Type 3 epithelial responses can be both adaptive and maladaptive, so the potential for therapeutic modulation exists in both directions. As noted above, aerosolized PRR ligands and Type 3 cytokines have the potential to augment resistance to microbial infections of the lungs, and some are in clinical trials for this purpose (e.g., NCT04312997). Conversely, in view of the multiple potential contributions of Type 3 responses to the pathogenesis of airway diseases, antioxidants, PRR antagonists, and cytokine antagonists are being studied in the mitigation of chronic airway diseases (Dudakov et al., 2015; Swedik et al., 2021). An important therapeutic principle for future studies will be disentangling beneficial short-term Type 3 responses from pathological chronic responses.

## Paracrine Signaling by Airway Epithelial Cells

When airway epithelial cells sense a pathogen and become activated, they signal both to neighboring epithelial cells and to leukocytes. Paracrine signaling activates a field of nearby epithelial cells to resist the local spread of infection, and is particularly well recognized for the release of IFNs in Type 1 responses (Schoggins, 2019). IFN stimulation of neighboring cells induces upregulation of many of the same ISGs that are upregulated cell-autonomously by viral PAMPs. In Type 2 and Type 3 responses, multiple secreted effector molecules also signal in a paracrine manner. For example, in Type 2 responses, chitinases and chitinase-like proteins have powerful signaling properties (Dickey, 2007; Przysucha et al., 2020), ClCa induces Muc5ac expression responses via MAPK13 signaling (Keeler et al., 2018), and secreted mucins signal via lectin interactions (Janssen et al., 2016). In Type 3 responses, many AMPs also have signaling properties (Lazzaro et al., 2020), such as cathelicidin that both promotes destruction of microbe lipoprotein membranes and is an agonist at the epidermal growth factor receptor. Similarly, ROS stimulate host immune responses in addition to directly inactivating pathogens (Kirkpatrick et al., 2018). Activated epithelial cells also communicate extensively with leukocytes, as illustrated in Figure 2, both to recruit leukocytes to sites of injury and/or pathogen invasion, and to help bias an appropriate leukocyte response. These interactions are the subject of multiple review articles (Frey et al., 2020; Hammad and Lambrecht, 2021), and are not further addressed here.

## Polarization of Airway Epithelial Immune Responses

We have discussed above how activation of airway epithelial cells by PAMPs, DAMPs, and cytokines leads to effector responses that are tailored to pathogen classes, consistent with the pathogen-selective epithelial responses first identified in *Drosophila* (Lemaitre et al., 1997; Tzou et al., 2000). Similar to the selectivity displayed in *Drosophila*, activation of mammalian airway epithelial cells with ODN-Pam2CSK4 or a bacterial lysate (Type 3 stimuli) resulted in no rise in IFN expression (Type 1 response) or in Muc5ac expression (Type 2 response) (Moghaddam et al., 2008; Cleaver et al., 2014).

In addition to the tailoring of responses, some evidence exists that activation of one type of epithelial response results in the suppression of other responses. For example, epithelial cells of subjects with allergic asthma (Type 2 immunity) have been found to have reduced interferon responses (Type 1 immunity) (Wark et al., 2005; Contoli et al., 2006). Mechanistically, induction of expression of the transcription factor Foxa3 by Type 2 stimuli enhances expression of a network of genes that inhibit IFN expression while promoting Muc5ac expression (Chen et al., 2014). Similarly, induction of expression of the Type 2 transcription factor SPDEF inhibited both Type 1 and Type 3 responses by direct interactions of SPDEF with the TLR adaptors TRIF and MyD88 (Korfhagen et al., 2012). Conversely, exposure to Type 3 stimuli found in farm dust suppresses Type 2 allergic airway inflammation through activation of the deubiquitinating enzyme A20 (Schuijs et al., 2015; Stein et al., 2016). Contrasting with these data is our finding that simultaneous administration of the Type 1 stimulus IFN-β and the Type 3 stimulus ODN-Pam2CSK4 did not suppress cytokine biomarkers of either response, and the antiviral effect of both together was additive (Goldblatt et al., 2020). In summary, interactions between distinct epithelial innate responses are complex and less well understood than polarizing responses in lymphocytes, so their successful therapeutic manipulation is likely to require further preclinical study.

## Discussion

In this article, we have reviewed the unique barrier properties of the airway epithelium, and the properties and mechanisms of its active sensing and defending against pathogens. Deeper understanding of these mechanisms may lead to further insight into causes of diseases and to the development of new therapeutics.

## Data Availability Statement

The original contributions presented in the study are included in the article/supplementary material, further inquiries can be directed to the corresponding author/s.

## Author Contributions

BD conceived the manuscript. All authors engaged in discussions and contributed to writing the article.

## Author Disclaimer

The views expressed are those of the author and not necessarily those of the NIHR or the Department of Health and Social Care.

## Conflict of Interest

SJ was an author on patents on the use of interferons for treatment of exacerbations of airway disease. SE, MT, and BD were inventors on US patent 8,883,174 “Compositions for Stimulation of Mammalian Innate Immune Resistance to Pathogens,” which has been licensed by their employer, the University of Texas MD Anderson Cancer Center, to Pulmotect, Inc., in which they hold equity. The remaining author declares that the research was conducted in the absence of any commercial or financial relationships that could be construed as a potential conflict of interest.

## Publisher’s Note

All claims expressed in this article are solely those of the authors and do not necessarily represent those of their affiliated organizations, or those of the publisher, the editors and the reviewers. Any product that may be evaluated in this article, or claim that may be made by its manufacturer, is not guaranteed or endorsed by the publisher.
